# INDIGENOUS UNDERSTANDINGS AND APPROACHES TO SUCCESSFUL AGING

**DOI:** 10.1093/geroni/igac059.1057

**Published:** 2022-12-20

**Authors:** Jordan Lewis, Steffi Kim

## Abstract

Indigenous peoples worldwide face unique challenges growing old. Many of these challenges are founded in historical colonization practices, present oppressive systems, racism, and underrepresentation in research, service provision, health education, and successful aging theories. The focus of this symposium is to discuss specific barriers and challenges related to Indigenous aging in rural and urban communities, as well as community strategies supporting aging well. The first presentation by Zayla Asquith-Heinz and colleagues will share the results of what successful aging or “Eldership” means in the Norton Sound southern subregion of Alaska. Results indicate that family plays a central role within the Norton Sound model of successful aging. The second presenter, Steffi Kim and Jordan Lewis, are discussing the role of cultural influences and Elder identity on successful aging in the context of Alaska Native Elders migrating from rural traditional communities to a western urban community. The third and fourth presentations by Sarah Russell and Rachel Quigley will share the results on what aging well means within Torres Strait Islander people. The results suggest that the availability and accessibility to traditional practices, language and foods can facilitate aging well within these communities. They will also describe the development and implementation of a toolbox of culturally appropriate screening tools and interventions. Lastly, Jordan Lewis will explore the Indigenous concept of “doing” successful aging rather than having good health. He will outline differences and similarities with BIPOC studies on successful aging.

